# Prepregnancy body mass index, gestational weight gain, and maternal prepartum inflammation in normal pregnancies: findings from a Chinese cohort

**DOI:** 10.1186/s12884-022-04849-y

**Published:** 2022-06-29

**Authors:** Kuanrong Li, Chuanzi Yang, Jiaying Fan, Xiaojun Li, Chongjuan Gu, Huishu Liu

**Affiliations:** 1 Clinical Epidemiology Group, Department of Clinical Research, Guangzhou Women and Children’s Medical Center, Guangzhou Medical University, 9 Jinsui Road, Guangzhou, 510623 China; 2 Department of Gynecology and Obstetrics, Guangzhou Women and Children’s Medical Center, Guangzhou Medical University, 9 Jinsui Road, Guangzhou, 510623 China

**Keywords:** Prepregnancy body mass index, Gestational weight gain, C-reactive protein, Systemic inflammation, Cohort study

## Abstract

**Background:**

Obesity has been linked to systemic inflammation in population studies.

**Objective:**

To examine the associations of prepregnancy body mass index (pBMI) and total gestational weight gain (tGWG) with maternal prepartum low-grade inflammation (LGI) and clinically significant inflammation (CSI) defined by serum C-reactive protein (CRP) concentration.

**Methods:**

Five thousand four hundred seventy-six Chinese women with uncomplicated pregnancies and recorded data on pBMI and prepartum body weight were included in this study. Blood samples were drawn before delivery for high-sensitivity CRP assay. Inadequate, optimal, and excessive tGWG were defined using the Institute of Medicine's recommendation. Multivariable Poisson regressions were used to estimate relative risks (RRs) for having prepartum LGI and CSI (defined as CRP concentration 3–10 and > 10 mg/L, respectively) across pBMI and tGWG categories.

**Results:**

The mean pBMI, mean tGWG, and median maternal prepartum CRP concentration were 20.4 kg/m^2^, 13.9 kg, and 3.3 mg/L, respectively. The prevalence of prepartum CSI and LGI was 7.2% and 47.8%. The adjusted RRs (95% confidence interval) of CSI for normal (18.5–24.9 kg/m^2^) and high (≥ 25 kg/m^2^) *vs.* low pBMI (< 18.5 kg/m^2^) were 1.35 (1.05–1.74) and 2.28 (1.53–3.39), respectively. The respective adjusted RRs of LGI were 1.19 (1.11–1.28) and 1.59 (1.42–1.77). The adjusted RRs for excessive *vs.* optimal tGWG was 1.18 (0.94–1.48) for CSI and 1.14 (1.07–1.21) for LGI.

**Conclusions:**

Prepregnancy overweight/obesity and excessive tGWG increase the risk of maternal prepartum systemic inflammation, which further highlights the importance of weight management before and during pregnancy.

**Supplementary information:**

The online version contains supplementary material available at 10.1186/s12884-022-04849-y.

## Background

C-reactive protein (CRP) is an acute-phase reactant synthesized by the hepatocytes in response to both infectious and non-infectious factors, and elevated CRP level is considered to reflect systemic inflammation [1, 2]. In general populations, positive associations have been found between increased adiposity and serum CRP concentration [3–6]. This supports the concept that obesity contributes to a state of low-grade systemic inflammation by promoting the production of a wide variety of pro-inflammatory cytokines [7].

The positive associations between prepregnancy body mass index (pBMI) and the risks of gestational diabetes and preeclampsia are well known [8–10], and both morbidities have been associated with elevated maternal serum CRP levels [11–13]. Several studies have also reported direct associations between prepregnancy obesity and elevated gestational CRP levels, and have suggested that inflammation is likely to be a mediator between prepregnancy obesity and adverse pregnancy outcomes [14–17].

It seems clear that women with prepregnancy overweight/obesity are prone to excessive gestational weight gain (GWG) [18–22]; it is thus of interest to examine whether excessive GWG, after taking into account prepregnancy adiposity, is an independent risk factor driving maternal systemic inflammation. Previous studies addressing this question have produced conflicting findings [23–25].

The goal of this study was to examine the associations of pBMI and total GWG (tGWG) with maternal prepartum serum CRP concentration in a large cohort of Chinese women who had undergone apparently healthy pregnancies.

## Methods

### Study population and data collection

This study was conducted at Guangzhou Women and Children’s Medical Center, and was based on a cohort initially including 22,455 pregnant women who delivered singletons following spontaneous pregnancy between 2016 and 2020. Data on pBMI, tGWG, and maternal prepartum serum high-sensitivity CRP concentration (measured on the same day of delivery or on the day preceding delivery) were available in these women. Exclusion criteria included women with the following characteristics: 1) pre-existing diabetes mellitus, hypertension, cardiovascular disease, renal diseases, liver diseases, thyroid diseases, or chronic infectious diseases (e.g. hepatitis B) (*n* = 5226); 2) preeclampsia, gestational diabetes mellitus, or any acute or chronic infectious diseases ascertained during the course of the index pregnancy (*n* = 9386); 3) premature rupture of membranes and placental problems such as placenta previa (*n* = 8925) in the index pregnancy; 4) fetal/neonatal structural abnormalities (*n* = 1085); 5) non-term (< 37 or ≥ 42 gestational weeks) deliveries (*n* = 903); 6) neonate 5-min Apgar score < 7 (*n* = 22); and 7) abnormal neonate birth weight (< 2500 or > 4000 g, *n* = 1329). We further excluded women with serum CRP concentration measured after delivery (*n* = 426) and women with seemingly erroneous values on body weight before delivery (< 30 or > 150 kg, *n* = 68) and on total gestational weight gain (determined by the lower and upper 0.5 percentiles, i.e., ≤ 0 or ≥ 29 kg, *n* = 181). After these exclusions, a total of 5476 women remained for final analysis. The study participant flowchart was provided in the Supplementary file.

Maternal demographics and reproductive history information as well as self-reported prepregnancy weight were collected at the antenatal booking visit. All women underwent ultrasound examinations performed at 11 ─ 13 gestational weeks. The crown-rump length from this ultrasound was used to determine gestational age. Maternal weight before delivery was obtained from the admission assessment form on labor and delivery. Maternal non-fasting venous blood samples, which were usually drawn shortly after admission, were collected in serum separator tubes and then analyzed in our laboratory. Serum CRP concentration was quantified using turbidimetric inhibition immunoassay kit (Strong Biotechnologies, Beijing, China).

This study was approved by the Guangzhou Women and Children’s Medical Center ethics committee (2021-030A01). Because this was a retrospective, chart review study, the ethics committee determined that patient informed consent was not required.

### Statistical analysis

Study participants' characteristics were described using mean and standard deviation for continuous variables and using frequency and percentage for categorical ones. The relationships of pBMI and tGWG with log-transformed maternal prepartum serum CRP concentration were analyzed using linear regression, with and without adjustment for other maternal and fetal characteristics. The linear models were extended with restricted cubic spline terms to identify possible non-linearity.

Maternal prepartum serum CRP concentration was then categorized into < 3 mg/L, 3 – 10 mg/L, and > 10 mg/L. This study defined the last two categories as low-grade inflammation (LGI) and clinically significant inflammation (CSI), consistent with other studies [6, 26–29]. Multivariable Poisson regression models were built to estimate the relative risks (RRs) of having LGI and CSI across pBMI and tGWG categories, which were defined using the WHO criteria (pBMI < 18.5, 18.5 – 24.9, and ≥ 25.0 kg/m^2^) and the approximate tertile cut-offs (tGWG < 12.0, 12.0—14.9, and ≥ 15.0 kg), respectively. The multivariable Poisson models adjusted for maternal age, parity, and gestational length. When applicable, pBMI and tGWG were mutually adjusted for. Test for trend was performed by substituting the actual CRP values with their median for each pBMI or tGWG category and then introducing the medians into the model as a continuous variable. Please note that women with prepartum CSI were excluded when modeling the risk of prepartum LGI.

For tGWG, we also adopted the GWG recommendation proposed by the Institute of Medicine (IOM) to categorize women into three groups, i.e., inadequate, optimal, and excessive GWG. According to the IOM recommendation, the normal tGWG is 12.5 – 18, 11.5 – 16, 7 – 11.5, and 5 – 9 kg for women with pBMI < 18.5, 18.5 – 24.9, 25 – 29.9, and ≥ 30 kg/m^2^, respectively [30]. Two additional Poisson regression models were built to examine whether nonoptimal tGWG is associated with different risks of LGI and CSI compared with optimal tGWG.

Two-sided *P* < 0.05 was considered statistically significant. The R software (version 4.0.2, R foundation, Vienna, Austria) was used to conduct the statistical analyses.

## Results

Among 5476 women, 394 (7.2%) and 2,617 (47.8%) met the criteria for CSI and LGI. The median prepartum CRP concentration for the entire cohort was 3.3 mg/L. Table 1 shows the baseline characteristics of the cohort. Women with prepartum CSI were statistically significantly younger, more likely to be nulliparous and to have higher pBMI than women without. The highest tertile of tGWG (≥ 15 kg) was more frequent among women with than without CSI (43.9% *vs.* 39.7%).

**Table 1:** Baseline characteristics of study participants, overall and by maternal prepartum CSI status

	Total (*n* = 5476)	Maternal prepartum CSI^a^		
		No (*n* = 5082)	Yes (*n* = 394)	*P* ^b^
Maternal age, mean (SD)	30.4 (4.0)	30.5 (4.0)	29.9 (3.9)	0.006
Parity category, n (%)				
Nulliparous	2512 (45.9)	2303 (45.3)	209 (53.0)	
Primiparous	2819 (51.5)	2645 (52.0)	174 (44.2)	
Multiparous	145 (2.6)	134 (2.7)	11 (2.8)	0.010
pBMI (kg/m^2^), mean (SD)	20.4 (2.5)	20.4 (2.5)	21.0 (3.0)	< 0.001
pBMI category, n (%)				
< 18.5 kg/m^2^	1193 (21.8)	1123 (22.1)	70 (17.8)	
18.5 – 24.9 kg/m^2^	3986 (72.8)	3696 (72.7)	290 (73.6)	
≥ 25 kg/m^2^	297 (5.4)	263 (5.2)	34 (8.6)	< 0.001
tGWG (kg), mean (SD)	13.9 (4.0)	13.8 (3.9)	14.0 (4.2)	0.664
tGWG tertiles, n (%)				
< 12 kg	1579 (28.8)	1461 (28.8)	118 (30.0)	
12 – 14.9 kg	1705 (31.2)	1602 (31.5)	103 (26.1)	
≥ 15 kg	2192 (40.0)	2019 (39.7)	173 (43.9)	0.075
Gestational length (weeks), mean (SD)	39.4 (0.9)	39.4 (0.9)	39.5 (0.9)	0.528

^a^Maternal prepartum CSI was defined as maternal prepartum serum CRP concentration > 10 mg/L

^b^*P* values were calculated by t-test for continuous variables and χ^2^ test for categorical variables

*CRP* C-reactive protein; *CSI* clinically significant inflammation; *pBMI* prepregnancy body mass index; *SD* standard deviation; *tGWG* total gestational weight gain

In both the unadjusted and adjusted linear regression analyses, pBMI and tGWG demonstrated statistically significant positive associations with maternal prepartum CRP concentration (Table 2). Restricted cubic spline analyses revealed no statistically significant non-linearity.

**Table 2:** Unadjusted and adjusted associations of pBMI and tGWG with log-transformed maternal prepartum serum CRP concentration (*n* = 5476)

	Unadjusted model		Adjusted model^a^		Adjusted RCS model^a^	
	β	*P*	β	*P*	β	*P*
pBMI	4.54 × 10^–2^	< 0.01	5.69 × 10^–2^	< 0.01	5.68 × 10^–2^	< 0.01
tGWG	1.40 × 10^–2^	< 0.01	1.69 × 10^–2^	< 0.01	1.28 × 10^–2^	0.297
pBMI RCS term					-1.85 × 10^–6^	0.985
tGWG RCS term					3.28 × 10^–5^	0.735

^a^pBMI and tGWG were mutually adjusted for in the multivariable models. The multivariable models were also adjusted for maternal age, parity, and gestational length

*CRP* C-reactive protein; *pBMI* prepregnancy body mass index; *RCS* restricted cubic spline; *tGWG* total gestational weight gain

The associations of pBMI and tGWG with maternal prepartum CSI and LGI risks are presented in Table 3. Compared with low pBMI (< 18.5 kg/m^2^), normal pBMI (18.5 – 24.9 kg/m^2^) and overweight/obesity (≥ 25.0 kg/m^2^) were statistically significantly associated with an increased risk of maternal prepartum CSI, with an RR (95% confidence interval [CI]) of 1.35 (1.05─1.74) and 2.28 (1.53─3.39), respectively (*P*_trend_ < 0.01), whereas tGWG tertiles showed no statistically significant associations. Overweight/obesity and normal BMI *vs.* low pBMI were also statistically significantly associated with an increased risk of maternal prepartum LGI [RR (95% CI): 1.19 (1.11─1.28), 1.59 (1.42─1.77); *P*_trend_ < 0.01]. For tGWG tertiles, a statistically significant positive association was seen for the highest (≥ 15.0 kg) *vs.* the lowest tertile (< 12.0 kg) [RR (95% CI): 1.17 (1.09─1.25)]. No statistically significant interaction was detected between pBMI and tGWG (data not shown).

**Table 3:** Adjusted associations of pBMI and tGWG with maternal prepartum CSI and LGI (*n* = 5476)

	No. prepartum inflammation/total no	RR (95% CI)^a^	*P*	*P*_trend_
*Maternal prepartum CSI (n* = *394)*^b^				
pBMI				
< 18.5 kg/m^2^	70/1193	1.00		
18.5 – 24.9 kg/m^2^	290/3986	1.35 (1.05–1.74)	0.020	
≥ 25.0 kg/m^2^	34/297	2.28 (1.53–3.39)	< 0.001	< 0.001
tGWG tertiles				
< 12.0 kg	118/1579	1.00		
12.0 – 14.9 kg	103/1705	0.83 (0.64–1.07)	0.150	
≥ 15.0 kg	173/2192	1.07 (0.86–1.35)	0.534	0.339
*Maternal prepartum LGI (n* = *2617)*^c^				
pBMI				
< 18.5 kg/m^2^	507/1123	1.00		
18.5 – 24.9 kg/m^2^	1935/3696	1.19 (1.11–1.28)	< 0.001	
≥ 25.0 kg/m^2^	175/263	1.59 (1.42–1.77)	< 0.001	< 0.001
tGWG tertiles				
< 12.0 kg	706/1461	1.00		
12.0 – 14.9 kg	790/1602	1.04 (0.96–1.12)	0.312	
≥ 15.0 kg	1121/2019	1.17 (1.09–1.25)	< 0.001	< 0.001

^a^pBMI and tGWG were mutually adjusted for in the multivariable models. The multivariable models also adjusted for maternal age, parity, and gestational length

^b^Maternal prepartum CSI was defined as maternal prepartum serum CRP concentration > 10 mg/L

^c^Maternal prepartum LGI was defined as maternal prepartum serum CRP concentration ≥ 3.0 and ≤ 10 mg/L. Women with prepartum CSI were excluded

*CI* Confidence interval, *CRP* C-reactive protein, *CSI* clinically significant inflammation, *LGI* low-grade inflammation, *pBMI* prepregnancy body mass index, *RR* relative risk, *tGWG* total gestational weight gain

As shown in Table 4, both excessive and inadequate tGWG *vs.* normal tGWG demonstrated positive but statistically non-significant associations with maternal prepartum CSI [RR (95% CI): 1.10 (0.88─1.39); 1.18 (0.94─1.48)]. Compared with normal tGWG, inadequate tGWG was associated with a statistically significantly decreased risk of maternal prepartum LGI risk [OR (95% CI): 0.91 (0.85–0.98)], while excessive tGWG showed a statistically significant but positive association [OR (95% CI): 1.14 (1.07–1.21)].

**Table 4:** Adjusted association of tGWG categorized by the IOM recommendation with maternal prepartum CSI and LGI (*n* = 5476)

	No. prepartum inflammation /total no	RR (95% CI)^a^	*P*
*Maternal prepartum CSI (n* = *394)*^b^			
Normal tGWG	177/2707	1.00	
Inadequate tGWG	104/1446	1.10 (0.88–1.39)	0.404
Excessive tGWG	113/1323	1.18 (0.94–1.48)	0.158
*Maternal prepartum LGI (n* = *2617)*^c^			
Normal tGWG	1277/2530	1.00	
Inadequate tGWG	618/1342	0.91 (0.85–0.98)	0.011
Excessive tGWG	722/1210	1.14 (1.07–1.21)	< 0.001

^a^The multivariable models adjusted for maternal age, parity, pBMI, and gestational length

^b^Maternal prepartum CSI was defined as maternal prepartum serum CRP concentration > 10 mg/L

^c^Maternal prepartum LGI was defined as maternal prepartum serum CRP concentration ≥ 3.0 and ≤ 10 mg/L. Women with prepartum CSI were excluded

*CI* Confidence interval, *CRP* C-Reactive protein, *CSI* Clinically significant inflammation, *IOM* Institute of Medicine, *LGI* Low-grade inflammation, *pBMI* Prepregnancy body mass index, *RR* Relative risk, *tGWG* Gestational weight gain;

## Discussion

In this study of women who had undergone apparently healthy pregnancies, pBMI and tGWG were both positively associated with maternal prepartum serum CRP concentration. Furthermore, higher pBMI was statistically significantly associated with increased prepartum CSI and LGI risks. Excessive tGWG, as defined by the IOM recommendation, was independently and statistically significantly associated with an increased LGI risk.

The association between obesity and maternal CRP level has been observed in pregnant women [14–17]. Although normal pregnancy is known as a process involving inflammation [31], elevated inflammation is considered pathogenic in the development of preeclampsia, retarded fetal growth, and many other maternal and fetal complications [31–33]. In order to identify elevated CRP levels, reference CRP intervals throughout normal pregnancies need to be established. In an ad hoc, small-scale Swedish study, the reference interval (i.e. the distance from 2.5 to 97.5 percentile) for maternal prepartum CRP concentration was 0.38 ─ 24.75 mg/L [34]. In our cohort, the corresponding interval was 0.56 ─ 16.7 mg/L. In one previous study including only 81 American women with normal pregnancy, the median CRP levels at or beyond 36 gestational weeks was 9 mg/L for women not in labor and 13 mg/L for women in labor [35]. In contrast, the median prepartum CRP concentration in the present study was considerably lower (3.3 mg/L), possibly owing to the low percentage of women with prepregnancy overweight or obesity. Despite a mean BMI of 25 kg/m^2^, another study reported remarkably low CRP concentrations at 36–38 gestational weeks across all BMI categories among normal pregnancies (e.g., the mean CRP concentration was lower than 1.69 mg/L even for women with BMI ≥ 30 kg/m^2^) [36], somewhat contradicting the current knowledge that pregnancy by nature is a low-grade inflammatory state.

Several studies investigating the longitudinal changes of maternal CRP concentration throughout pregnancy have reported either increasing or decreasing trends [34, 37]. It is interesting that in the study by Larsson et al.[34], a notable decrease in maternal CRP concentration was seen prepartum (0–14 days before delivery) after an overall increasing trend until the 34 ─ 38 gestational weeks. Although our study measured maternal CRP on one single occasion when the CRP concentration is supposed to be low, we ascertained 47.8% women with LGI, confirming the extensive involvement of inflammation throughout pregnancy [31]. This study also suggests that the proinflammatory effect of prepregnancy adiposity persists as pregnancy proceeds, providing a possible explanation to the high CRP concentration in the colostrum from obese mothers and the presence of CRP in breast milk as a result of elevated maternal postpartum CRP concentration [38, 39]. However, this study is inconsistent with the study by Friss CM et al., where they found that the evident association between adiposity and maternal CRP concentration diminished toward the end of pregnancy [36].

Evidence obtained from non-pregnant populations indicates a positive association between weight gain and systemic inflammation characterized by elevated CRP concentration [3, 4, 40, 41]. In this study, we observed a similar positive association between tGWG and maternal prepartum CRP concentration, consisting with one prior study, in which per 1-kg increment in GWG was associated with a 3% increase in maternal CRP concentration [25]. However, the association was null in the study by Friss CM et al.[36]. In another study, tGWG showed positive associations with adiponectin and leptin but not with CRP [23].

Although we reported a positive association of tGWG with maternal prepartum CRP concentration and an increased LGI risk for women with excessive GWG, we could not determine a clear causal relationship, given the findings from prospective investigations suggesting that higher baseline CRP levels might promote the future weight gain [3, 42–44]. In order to rule out this reverse causality, prepregnancy or early-pregnancy CRP concentration should also be taken into account.

Our study’s strengths include its relatively large sample size and careful selection of only pregnancies without significant medical comorbidities. However, a few limitations need to be acknowledged. First, this study lacked some important data. As we discussed above, baseline maternal CRP concentration was not available in this study; thus we were unable to confirm the causal relationship between tGWG and maternal prepartum CRP level. We were also unable to adjust for some lifestyle factors in our multivariable analyses due to lack of data. For example, information on cigarette smoking and alcohol consumption was not collected, although they are believed to be relatively uncommon among Chinese women. In addition, we did not collect any data on other confounding factors including socioeconomic status, diet, and physical activity. According to previous studies [45–47], these factors might affect serum CRP concentration in non-pregnant populations, but we could not rule out their potential confounding effects in this study. Secondly, we followed the protocols of several previous studies to define LGI and CSI [26, 48, 49], but there is no clear consensus. In addition, as those studies measured CRP concentration in non-pregnant populations, and therefore their protocols might not be suitable for this study due to the typically elevated serum CRP levels in pregnant women. Thirdly, pBMI based on self-reported prepregnancy body weight was subject to recall bias and how it biased our results was determined by the pattern of misreporting. Lastly, the present study was conducted in a considerably lean cohort, which would limit the generalization of our results to populations where overweight and obesity are much more prevalent.

## Conclusions

This cohort study among Chinese women with uncomplicated pregnancies suggests that higher pBMI and excessive tGWG may increase the risk of maternal prepartum systemic inflammation, further highlighting the importance of weight management before and during pregnancy.

## Supplementary information

**Additional file 1.**

## Data Availability

The data that support the findings of this study are available from the corresponding authors upon reasonable request.
