# Supplementary figures and images for: Prepregnancy body mass index, gestational weight gain, and maternal prepartum inflammation in normal pregnancies: findings from a Chinese cohort
Source: BMC Pregnancy Childbirth. 2022 Jun 29;22:531. doi: 10.1186/s12884-022-04849-y (PMC9245225; doi:10.1186/s12884-022-04849-y)

## Study Participant Flowchart

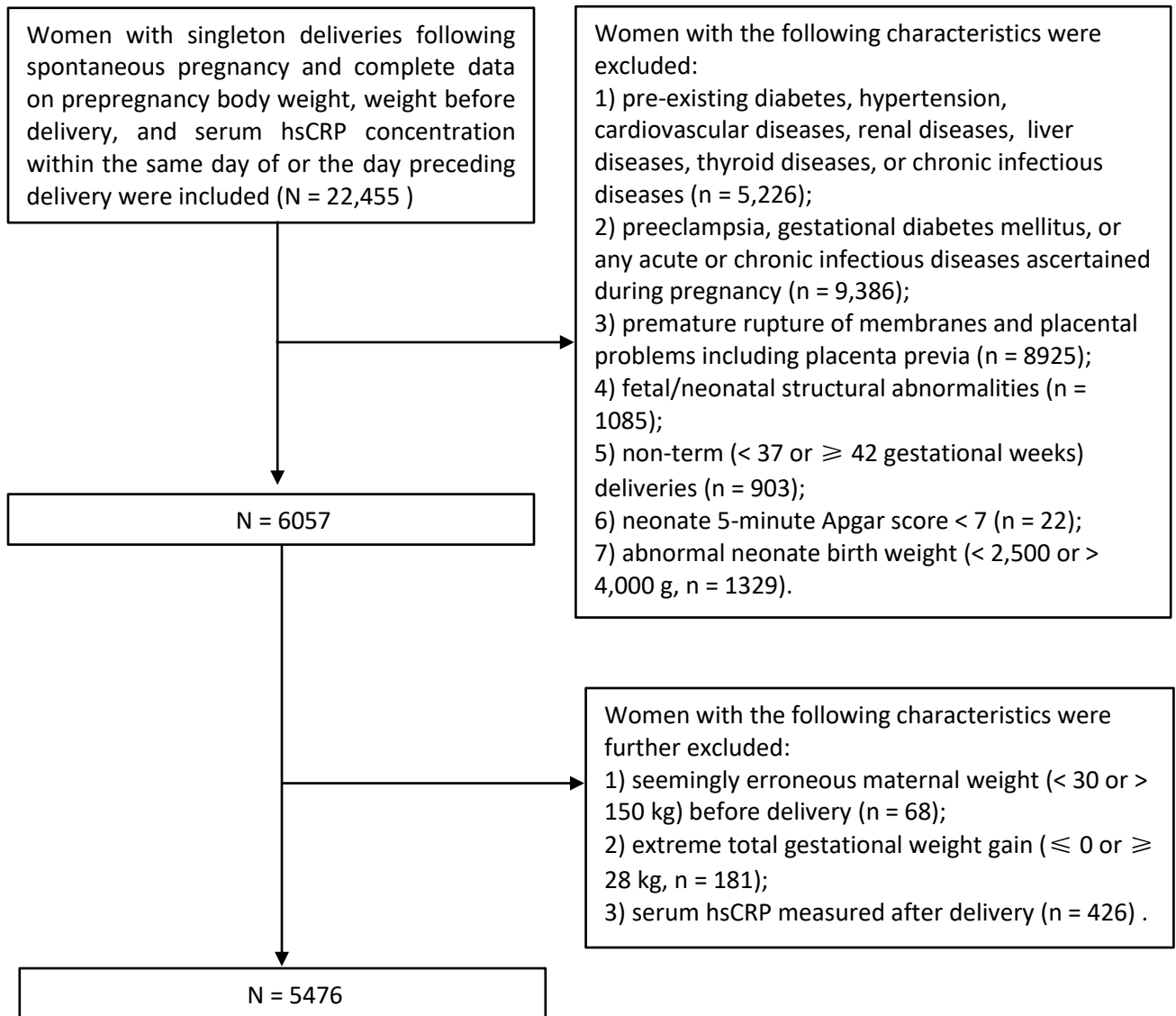

Supplement: Supplementary file 1 — Additional file 1. [file 12884_2022_4849_MOESM1_ESM.pdf]
